# IAA Plays an Important Role in Alkaline Stress Tolerance by Modulating Root Development and ROS Detoxifying Systems in Rice Plants

**DOI:** 10.3390/ijms232314817

**Published:** 2022-11-26

**Authors:** Changkun Ma, Shuai Yuan, Biao Xie, Qian Li, Quanjiu Wang, Mingan Shao

**Affiliations:** 1 State Key Laboratory of Eco-Hydraulics in Northwest Arid Region, Xi’an University of Technology, Xi’an 710048, China; 2 College of Horticulture, Northwest A & F University, Xianyang 712100, China; 3 Key Laboratory of Ecosystem Network Observation and Modeling, Institute of Geographic Sciences and Natural Resources Research, Chinese Academy of Sciences, Beijing 100101, China

**Keywords:** alkaline stress, auxin, ROS detoxifying mechanism, rice (*Oryza sativa*)

## Abstract

Auxin regulates plant growth and development, as well as helps plants to survive abiotic stresses, but the effects of auxin on the growth of alkaline-stressed rice and the underlying molecular and physiological mechanisms remain unknown. Through exogenous application of IAA/TIBA, this study explored the physiological and molecular mechanisms of alkaline stress tolerance enhancement using two rice genotypes. Alkaline stress was observed to damage the plant growth, while exogenous application of IAA mitigates the alkaline-stress-induce inhibition of plant growth. After application of exogenous IAA to alkaline-stressed rice, dry shoot biomass, foliar chlorophyll content, photosynthetic rate in the two rice genotypes increased by 12.6–15.6%, 11.7–40.3%, 51.4–106.6%, respectively. The adventitious root number, root surface area, total root length and dry root biomass in the two rice genotypes increased by 29.3–33.3%, 26.4–27.2%, 42.5–35.5% and 12.8–33.1%, respectively. The accumulation of H_2_O_2_, MAD were significantly decreased with the application of IAA. The activities of CAT, POD, and SOD in rice plants were significantly increased by exogenous application of IAA. The expression levels of genes controlling IAA biosynthesis and transport were significantly increased, while there were no significant effects on the gene expression that controlled IAA catabolism. These results showed that exogenous application of IAA could mitigate the alkaline-stress-induced inhibition of plant growth by regulating the reactive oxygen species scavenging system, root development and expression of gene involved in IAA biosynthesis, transport and catabolism. These results provide a new direction and empirical basis for improving crop alkaline tolerance with exogenous application of IAA.

## 1. Introduction

As the world’s most important cereal, rice is consumed by more than three billion people worldwide and provides 50 to 80% of the calories they require each day. Globally, rice growth and production are affected by various environmental factors, among which soil saline-alkalization stress is one of the main stress factors [1]. According to the world soil map, approximately 434 million hectares of soil suffer from alkalinity [2]. Moreover, soil alkalization has been reported to be increasing as a result of natural events and human interaction, such as weathering of parental rocks that release soluble salts, wind and rain that deposit oceanic salts, and irrigation that contains trace amounts of sodium chloride. [1,3]. In order to solve the soil saline-alkaline problem, several measures have been applied in recent years, such as breeding crops that can grow on saline-alkaline soils, soil amelioration, etc. For soil amelioration measurements, flooding fields is one of the most important strategies for alleviating the damage caused by saline-alkaline stress on plants by leaching sodium, magnesium, and calcium into the groundwater [4,5]. However, these strategies will result in another problem, a decreased Na^+^ concentration and a high pH in the soil [6]. The low concentration of Na^+^ and high pH in soil (alkaline stress) also affect the growth and production of plants by disrupting ion uptake and causing oxidative stress and osmotic stress in plant cells. However, little attention has been paid to the response of plants to low soil Na^+^ concentrations and high soil pH conditions. Despite that, understanding how plants respond to low Na^+^ concentrations and high soil pH conditions is crucial to understanding saline-alkaline tolerance mechanisms.

Plants can re-program several defense responses when they are exposed to environmental stresses. To minimize Na^+^ toxicity during saline-alkaline, plants have developed several strategies, including limiting Na^+^ absorption, encapsulating toxic Na^+^ in vacuoles, and limiting Na^+^ transport to shoots [3]. In rice, for example, saline tolerance was enhanced by a transporter, OsHKT1;5, that can withdraw Na^+^ from the vasculature [7]. Despite Na^+^ toxicity, plants subjected to saline-alkaline stress also experience osmotic stress. Therefore, plants equipped with the capacity to tolerate osmotic stress may increase their tolerance to saline-alkaline stress. It has been demonstrated that the build-up of suitable osmolytes within the cytosol plays an important role in osmotic stress tolerance, since water intake by plants was decreased in an environment in which the soil’s osmotic potential was elevated [3]. The increase in antioxidant enzyme activity is another mechanism by which plants adapt to saline-alkaline stresses. It has been shown that saline-alkaline tolerant rice [8], tomato [9] and *Trifolium alexandrinum* L. [10] are better protected from reactive oxygen species (ROS) as a result of an increase in antioxidant enzyme activity in response to salt-alkaline stresses. In addition, saline-alkaline stress can increase nutritional stress in plants, since a high pH in the soil can affect the availability of minerals, such as nitrogen and iron. Thus, plants have developed mechanisms to deal with the nutritional stress caused by the high pH of the saline-alkaline soil. It has been shown in several studies that a high iron acquisition rate, nitrate assimilation rate, and calcium metabolism are necessary for plants to adapt to salt-alkaline stress [5,11,12]. Furthermore, the accumulation of organic acids and the secretion of H^+^ are important mechanisms that equip plants with saline-alkaline stress resistance [11,13].

Auxin (IAA, indole-3-acetic acid) is one of the most important plant hormones that regulate plant growth and development, including the morphogenesis of shoots and roots and their elongation [14]. In addition, recent studies have shown that auxins are also important in allowing plants to tolerate various abiotic stresses, including salt stress [15], sodic alkaline stress [16], and phosphorus-deficient [17]. When abiotic stress conditions exist, auxin homeostasis is disturbed and as a consequence, processes and signal transduction governed by auxin are impacted. For instance, when auxin homeostasis is disrupted through alkaline stress, root growth is inhibited [16,18], while a higher concentration of endogenous IAA can potentially promote the root growth of plants by modulating genes involved in auxin biosynthesis, efflux, and conjugation and degradation [15,19].It has been demonstrated that the application of IAA/NAA (naphthylacetic acid) increases the root growth of plants, such as rice [20], maize [21], citrus rootstock [18] and *malus* rootstocks [19], under abiotic stress conditions. Recently, several studies have revealed the mechanisms through which auxin enhances plant stress resistance. Naser and Shani (2016), for example, provide an overview of the mechanism by which auxin promotes plant growth and development under conditions of osmotic stress [14]. They demonstrated that auxin homeostasis was modulated by regulating the expression of genes involved in auxin biosynthesis (*YUC*, *TAA1*), transport (*PIN*), perception (*TIR*/A*FB*, *Aux*/*IAA*), and inactivation/conjugation (*GH3*, *miR167*, *IAR3*) [14]. The stress-modulated auxin gradient, in turn, drives a wide range of physiological and developmental processes, including the opening of stomata, the production of aquaporin, the positioning of lateral roots [14], and the accumulation of osmoregulation substances, such as proline and soluble sugar [22,23]. Furthermore, auxin contributes to the stress tolerance of plants through the scavenging of reactive oxygen species, the decrease of Na^+^ accumulation and protection of the photosystem II (PSII) from damage [16,24]. As an example, Gong et al. (2014) observed that exogenous application of IAA increased the concentration of IAA in cucumber leaves, thereby enhancing the activity of CAT to alleviate sodic alkaline stress [16]. As well as the mechanisms outlined above, stress-modulated auxin gradients are also involved in changing root parameters, which are also involved in stress resistance mechanisms [15,25]. Saini et al. (2021), for instance, concluded that higher levels of auxin in rice roots stimulated the development of root systems, ultimately resulting in salt-tolerant genotypes of rice [15].

According to our previous studies, Dongdao-4 rice genotype is not only more tolerant to saline-alkaline stress [11] but is also more tolerant to low Na^+^ concentrations and high soil pH levels (alkaline stress) than Jigeng-88 rice genotype (unpublished paper). In addition, we found that alkaline stress reduced IAA concentrations in Dongdao-4 plants less than those in Jigeng-88 plants, suggesting that IAA may play an important role in alkaline stress tolerance of Dongdao-4 rice genotype. Therefore, to clarify the mechanisms involved in the adaptation of rice plants to alkaline stress, we undertook a comparative study of the effects of exogenously applied IAA and TIBA (2,3,5-triiodobenzoic acid, which inhibits the transport of auxins) on the growth of these two rice genotypes under alkaline stress.

## 2. Results

### 2.1. Dongdao-4 Seedlings Are More Tolerant to Alkaline Stress than Jigeng-88 Seedlings

In order to assess the differences in alkaline stress resistance between Dongdao-4 and Jigeng-88 plants, a solution supplemented with 20 mM NaHCO_3_ was applied for 2 days to one-week-old rice seedlings of both rice genotypes. The effects of alkaline stress on plant growth and survival were observed. As shown in Figure S1, Dongdao-4 plants had significantly higher survival rates after being exposed to alkaline stress than Jigeng-88 plants. In addition, we compared some key physiological processes between these two genotypes of rice and found that in comparison with Jigeng-88 seedlings, Dongdao-4 seedlings are more resistant to alkaline stress (unpublished paper).

### 2.2. Effect of Alkaline Stress on Auxin Concentration

**Figure 1:**
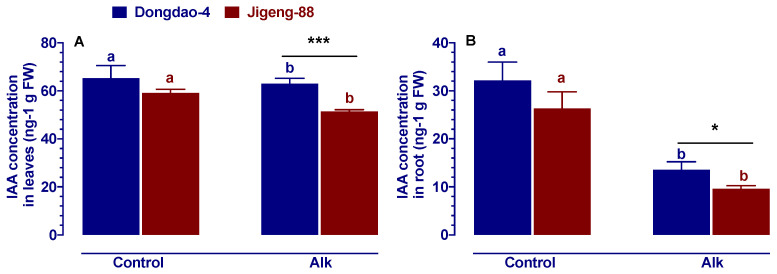
IAA concentrations of (**A**) shoots and (**B**) roots of Dongdao-4 and Jigeng-88 seedlings grown in control and alkaline stress medium. Two-week-old rice seedlings cultivated in normal culture solution were moved to culture solution that was added with 20 mM NaHCO_3_ and had a pH of 8.5 for 5 days. Alk = alkaline stress. Bars = 10 cm. Data are means ± SE (n ≥ 5). Means with different letters are significantly different between control and alkaline stress of the same genotype (*p* < 0.05). Asterisks indicate significant differences within the same treatment (* *p* < 0.05, *** *p* < 0.001).

As auxin plays a crucial role in salt tolerance, we hypothesized that auxin may also play a significant role in alkaline tolerance and that the composition of IAA in plant tissues may also differ among plants with different levels of alkaline tolerance. In order to investigate this hypothesis, we measured the concentration of IAA in the shoots and roots of Dongdao-4 and Jigeng-88 plants grown on both control and alkaline media (Figure 1). According to Figure 1, we can see that there were no differences in IAA concentrations between the two genotypes of rice under control conditions. However, when both rice genotypes were exposed to alkaline stress, both Jigeng-88 and Dongdao-4 exhibited significant reductions in their levels of IAAs. Under alkaline treatment, shoot IAA concentrations were significantly reduced by 3.4% and 13.1%, respectively, compared to the control treatment for Dongdao-4 and Jigeng-88 plants; the decreased values of root IAA concentrations for Dongdao-4 and Jigeng-88 were 57.7% and 63.5%, respectively. Those results may indicate that in rice plants, IAA plays a major role in the tolerance of alkaline stress.

### 2.3. Effect of IAA and TIBA on Plant Growth under Alkaline Stress

**Figure 2:**
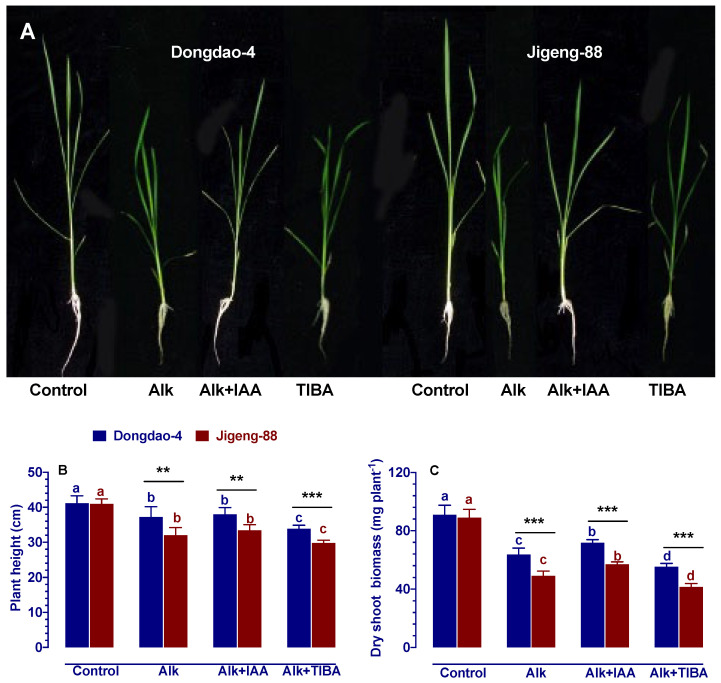
Effects of IAA and TIBA on (**A**) symptoms, (**B**) plant height, (**C**) dry shoot biomass of Dongdao-4 and Jigeng-88 seedlings after treatment with alkaline stress. Treatments were as described in Figure 1. Means with different letters are significantly different (*p* < 0.05) between control and “Alk” and “Alk + IAA” and “Alk + TIBA” of the same genotype. Asterisks indicate significant differences between different genotypes within the same treatment (** *p* < 0.01, *** *p* < 0.001).

In order to evaluate the effect of IAA on rice’s ability to tolerate alkaline conditions, the performance of the Dongdao-4 and Jigeng-88 plants was compared with the application of both IAA and TIBA. As shown in Figure 2, no growth differences were observed between these two genotypes in the control solution. While undergoing alkaline treatment, the growth of both rice genotypes was suppressed. Under alkaline stress, Dongdao-4 plants experienced a much smaller amount of suppression than Jigeng-88 plants, which resulted in a significantly higher height of the plants and dry shoot biomass in Dongdao-4 seedlings than in Jigeng-88 seedlings (Figure 2B,C).

As compared to plants under alkaline stress, exogenously applied IAA significantly increased dry shoot biomass by 12.6% and 15.6%, respectively, in the Dongdao-4 and Jigeng-88 plants (Figure 2C), which indicates that exogenously applied IAA significantly reduced the inhibitory effects of alkaline stress on plant growth in both rice genotypes (Figure 2C). In contrast, the growth-inhibiting effects of alkaline stress on rice plants were significantly enhanced by exogenous application of TIBA as evidenced by lower plant height and dry shoot biomass compared to alkaline stress (Figure 2B,C). Compared with the alkaline treatment, the plant height of Dongdao-4 and Jigeng-88 plants treated with TIBA decreased significantly by 9.0% and 6.9%, respectively; dry shoot biomass decreased significantly by 13.3% and 18.1%, respectively, in Dongdao-4 and Jigeng-88 plants (Figure 2B,C).

### 2.4. Effect of IAA and TIBA on Foliar Chlorophyll Concentration and Photosynthetic Rates under Alkaline Stress

**Figure 3:**
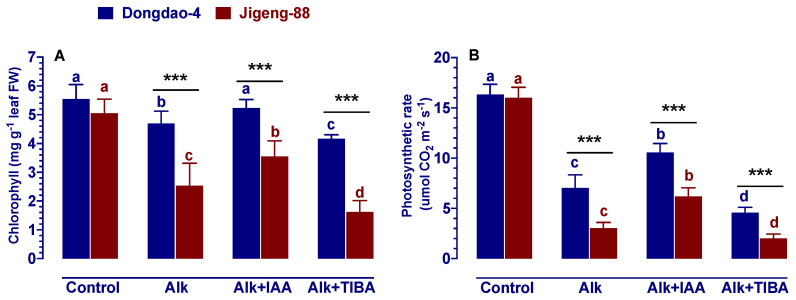
Effects of IAA and TIBA on (**A**) foliar chlorophyll concentration and (**B**) photosynthetic rates of Dongdao-4 and Jigeng-88 seedlings after treatment with alkaline stress. Treatments were as described in Figure 1. Means with different letters are significantly different (*p* < 0.05) between control and “Alk” and “Alk + IAA” and “Alk + TIBA” of the same genotype. Asterisks indicate significant differences between different genotypes within the same treatment (*** *p* < 0.001).

The chlorophyll concentration and photosynthetic rate of both genotypes were significantly reduced when exposed to alkaline stress, and the amount of the decrease for Jigeng-88 plants was greater than that for Dongdao-4 plants (Figure 3). In terms of foliar chlorophyll concentration, it was found that there was no difference between control and exogenous application of IAA for Dongdao-4 seedlings, however, the foliar chlorophyll concentration of Jigeng-88 seedlings after treatment with IAA is still 29.8% lower than the control condition (Figure 3A). As for photosynthetic rates, exogenous application of IAA alleviated the suppressive effects of alkaline stress for both genotypes, with Dongdao-4 plants showing significantly higher photosynthetic rates than Jigeng-88 plants (Figure 3B). Following the application of TIBA treatment, the foliar chlorophyll concentration and photosynthetic rate of both rice genotypes were significantly reduced, with the decrease being more pronounced for Jigeng-88 plants than for Dongdao-4 plants (Figure 3B).

### 2.5. Effect of IAA and TIBA on Root System Architecture under Alkaline Stress

**Figure 4:**
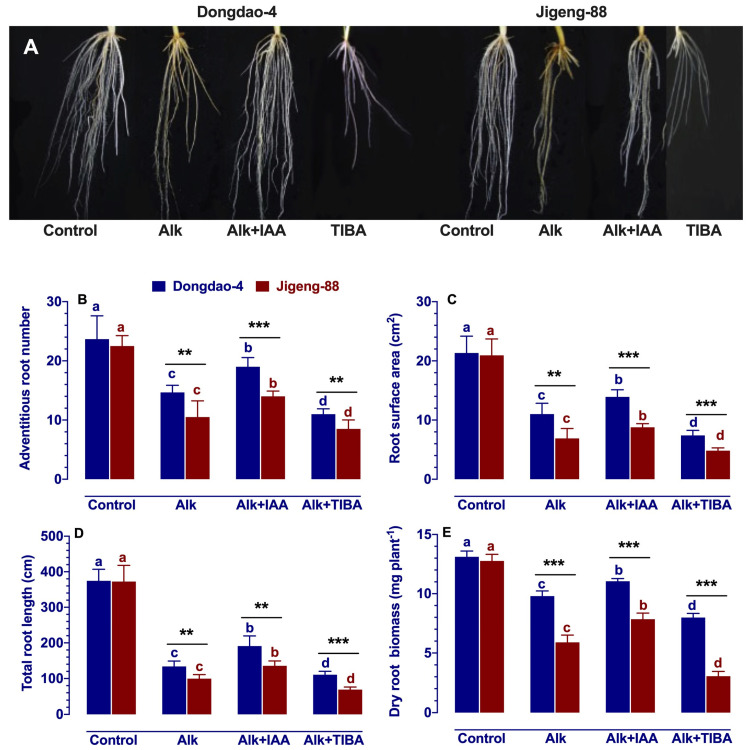
Effects of IAA and TIBA on (**A**) root symptoms, (**B**) adventitious root number, (**C**) root surface area, (**D**) total root length, (**E**) dry root biomass of Dongdao-4 and Jigeng-88 seedlings after treatment with alkaline stress. Treatments were as described in Figure 1. Means with different letters are significantly different (*p* < 0.05) between control and “Alk” and “Alk + IAA” and “Alk + TIBA” of the same genotype. Asterisks indicate significant differences between different genotypes within the same treatment (** *p* < 0.01, *** *p* < 0.001).

Considering the importance of the root system for nutrient uptake and its relationship with growth hormones, we investigated the effects of IAA and TIBA on root structure under alkaline stress. As shown in Figure 4, the root systems of the two genotypes developed similarly in the control solution, but alkaline treatment significantly reduced root parameters in both genotypes, with a greater reduction in Jigeng-88 plants than in Dongdao-4 plants. As a result, Dongdao-4 plants under alkaline stress had a significantly higher number of adventitious root number, root surface area, dry root biomass, and total root length than Jigeng-88 plants (Figure 4B–E). The exogenous application of IAA reversed the inhibition of root growth caused by alkaline in both rice genotypes. As shown in Figure 4B–E, when IAA was applied, adventitious roots, root surfaces, root lengths, and root biomass of Dongdao-4 plants were significantly increased compared with the alkaline treatment by 29.3%, 26.4%, 42.5%, and 12.8%, respectively; the adventitious root number, root surfaces, root lengths, and root biomass of Jigeng-88 plants increased significantly by 33.3%, 27.2%, 35.5%, and 33.1%, respectively. When TIBA was applied under alkaline stress conditions to both rice genotypes, the root parameters of both rice genotypes significantly decreased, and adventitious root numbers, root surface area, total root length, and dry root biomass in Dongdao-4 plants were significantly higher than those in Jigeng-88 plants (Figure 4B–E).

### 2.6. Effect of IAA and TIBA on the Content of H_2_O_2_ and Malondialdehyde under Alkaline Stress

**Figure 5:**
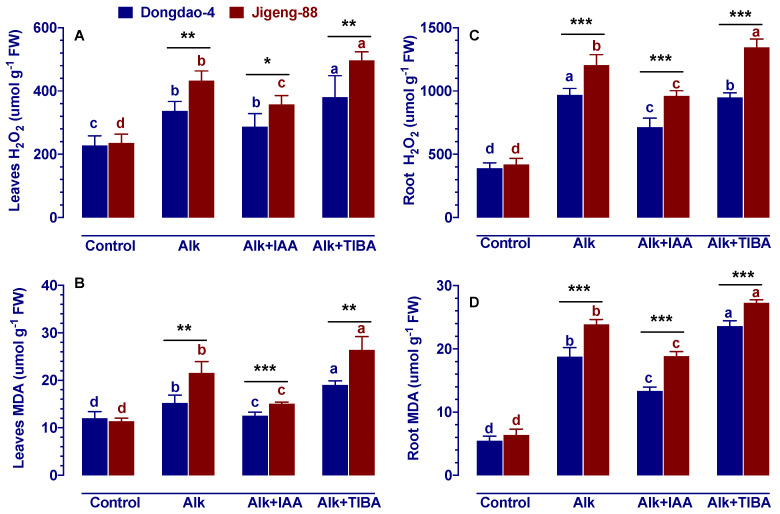
Effects of IAA and TIBA on contents of (**A**,**C**) H_2_O_2_, (**B**,**D**) malondialdehyde (MDA) of Dongdao-4 and Jigeng-88 seedlings after treatment with alkaline stress. Treatments were as described in Figure 1. Means with different letters are significantly different (*p* < 0.05) between control and “Alk” and “Alk + IAA” and “Alk + TIBA” of the same genotype. Asterisks indicate significant differences between different genotypes within the same treatment (* *p* < 0.05, ** *p* < 0.01, *** *p* < 0.001).

Whenever plants are subjected to abiotic stress, they often demonstrate oxidative stress symptoms, such as increased levels of reactive oxygen species (ROS) and malondialdehyde (MDA). Accordingly, MDA and H_2_O_2_ levels were measured under alkaline stress conditions with the addition of IAA and TIBA. As shown in Figure 5, alkaline stress significantly increases H_2_O_2_ and MDA content, with the increase being greater in Jigeng-88 seedlings than in Dongdao-4 seedlings. As a result, Jigeng-88 seedlings showed significantly higher levels of H_2_O_2_ and MDA in their leaves and roots as compared with Dongdao-4 seedlings. For both rice genotypes, exogenous application of IAA reduced alkaline-induced increases in H_2_O_2_ and MDA content. However, the decrease in root H_2_O_2_ and MDA was more pronounced in Dongdao-4 plants (26.4%, 62.0%) than in Jigeng-88 plants (20.2%, 22.6%) (Figure 5). When exogenous TIBA was applied to these two rice genotypes, a significant increase in H_2_O_2_ and MDA levels was observed in the leaf and root tissues under alkaline stress conditions, with the increase being larger for Jigeng-88 seedlings than for Dongdao-4 seedlings. Consequently, the levels of H_2_O_2_ and MDA in Jigeng-88 seedlings were higher than those in Dongdao-4 seedlings when TIBA was applied.

### 2.7. Effect of IAA and TIBA on Activities of Antioxidant Enzymes under Alkaline Stress

**Figure 6:**
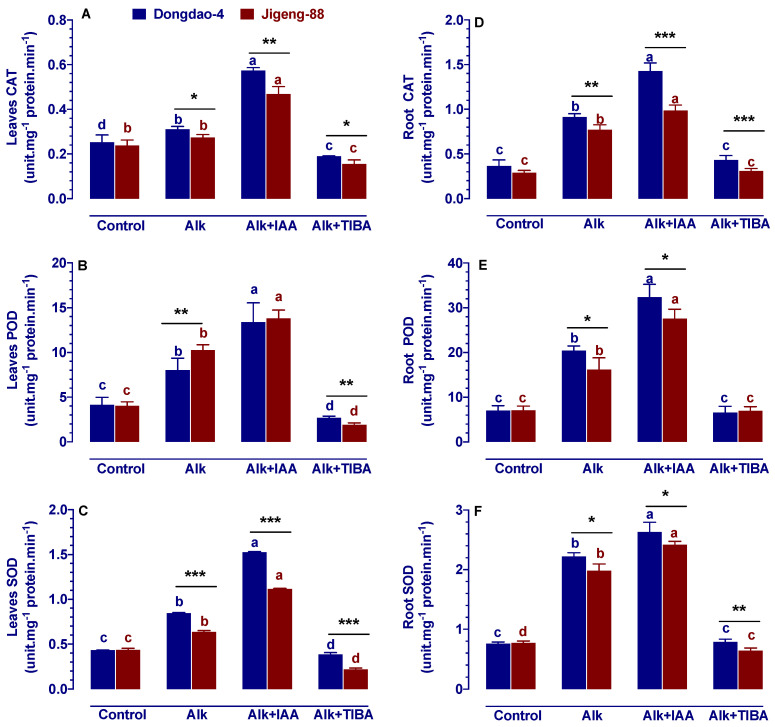
Effects of IAA and TIBA on antioxidant enzymes in shoots/roots of Dongdao-4 and Jigeng-88 seedlings after treatment with alkaline stress. (**A**,**D**) Catalase (CAT) in leaves/roots. (**B**,**E**) Peroxidase (POD) in leaves/roots. (**C**,**F**) Superoxide dismutase (SOD) leaves/roots. Treatments were as described in Figure 1. Means with different letters are significantly different (*p* < 0.05) between control and “Alk” and “Alk + IAA” and “Alk + TIBA” of the same genotype. Asterisks indicate significant differences between different genotypes within the same treatment (* *p* < 0.05, ** *p* < 0.01, *** *p* < 0.001).

According to our previous findings, Dongdao-4 seedlings accumulate less H_2_O_2_ and MDA under alkaline stress than Jigeng-88 seedlings, which led us to examine the activities of the major antioxidant enzymes in the two rice genotypes. Our results showed that under the control conditions, Dongdao-4 and Jigeng-88 plants had similar levels of CAT, POD, and SOD enzyme activity, except for root CAT, where Dongdao-4 plants had a significantly higher level of root CAT activity than Jigeng-88 plants (Figure 6). When rice seedlings were exposed to alkaline stress, the activity of CAT, POD, and SOD increased significantly, and the increased activity of these enzymes was significantly higher in Dongdao-4 plants when compared to Jigeng-88 plants. As a result, the activities of CAT, POD, and SOD in Dongdao-4 plants were significantly greater than those of Jigeng-88 plants under alkaline stress (Figure 6). When exogenous application of IAA under alkaline stress conditions was conducted, significant increases in the activity of CAT, POD, and SOD were observed in both rice genotypes. It was found that the magnitude of the increase in levels of CAT and SOD was significantly greater in Dongdao-4 plants (83.9% and 82.1%) than in Jigeng-88 plants (71.1% and 75%), which led to significantly higher levels of CAT and SOD in the leaves of Dongdao-4 plants than in Jigeng-88 plants (Figure 6A,C). Although IAA treatment-induced increase in root CAT, POD, and SOD was more pronounced in Jigeng-88 plants (28.6%, 75.0% and 22.2%) than in Dongdao-4 plants (57.1%, 82.1% and 18.5%), the activities of root CAT, POD, and SOD in Dongdao-4 plants was still significantly higher than that in Jigeng-88 plants (Figure 6D–F). The exogenous application of TIBA under conditions of alkaline stress significantly reduced the activities of these enzymes, and it was found that the TIBA-induced reduction in Jigeng-88 plants was significantly higher than that in Dongdao-4 plants, leading to a significantly greater content of CAT, POD and SOD in the leaves and roots of Dongdao-4 plants as compared with Jigeng-88 plants (Figure 6A–D,F). Additionally, it should be noted that there was no significant difference in root activity of these enzymes between Dongdao-4 and Jigeng-88 plants when TIBA was applied (Figure 6E).

### 2.8. Effect of Alkaline Stress on the Genes Involved in IAA Biosynthesis, Transport and Catabolism

**Figure 7:**
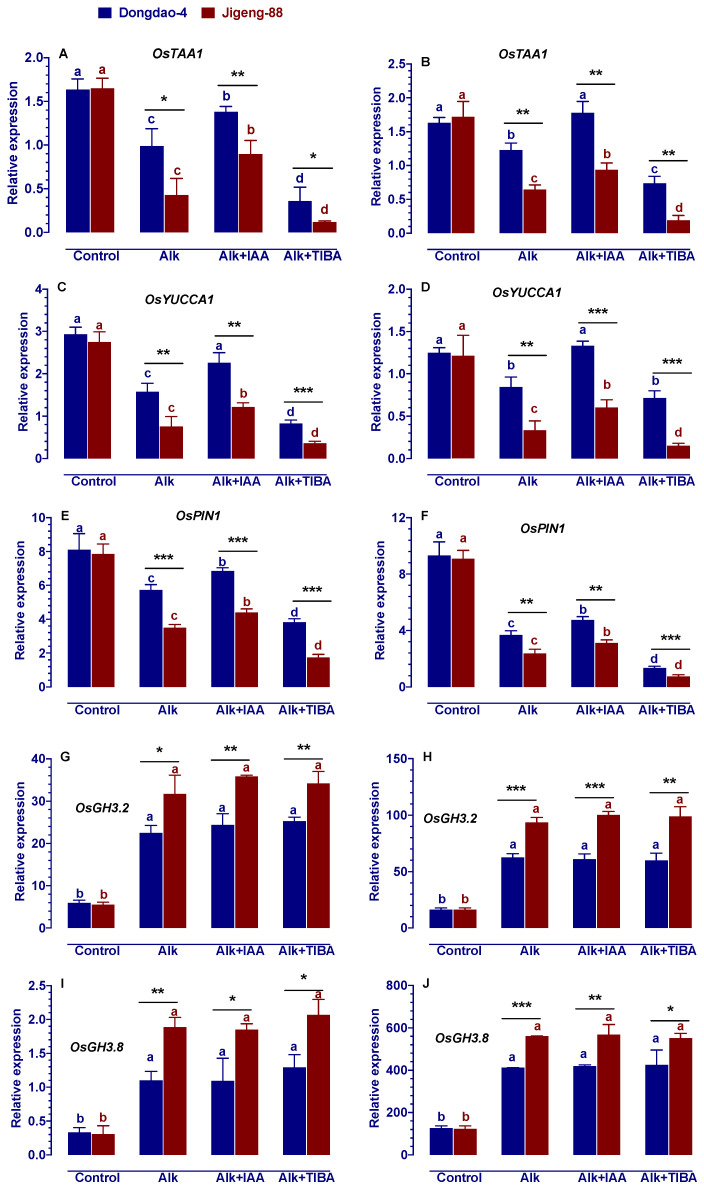
Effects of alkaline stress on the expression of genes involved in IAA biosynthesis (O*sTAA1* and *OsYUCCA1*), transport (*OsPIN1*) and catabolism (*OsGH3.2* and *OsGH3.8*) in shoots (**A**,**C**,**E**,**G**,**I**) and roots (**B**,**D**,**F**,**H**,**J**) of Dongdao-4 and Jigeng-88 seedlings. Treatments were as described in Figure 1. Means with different letters are significantly different (*p* < 0.05) between control and “Alk” and “Alk + IAA” and “Alk + TIBA” of the same genotype. Asterisks indicate significant differences between different genotypes within the same treatment (* *p* < 0.05, ** *p* < 0.01, *** *p* < 0.001).

In this study, it was found that alkaline stress significantly affected the expression of genes controlling the synthesis, transportation, and catabolism of IAA in both Dongdao-4 and Jiigeng-88 plants. A significant reduction was observed in the expression levels of O*sTAA1*, *OsYUCCA1* and *OsPIN1*, which controlling IAA biosynthesis and transport, respectively, (Figure 7A–F), while a significant increase in the expression levels of IAA catabolism genes, *OsGH3.2* and *OsGH3.8*, was observed (Figure 7G–J) when rice plants are subject to alkaline stress. As compared to Dongdao-4, treatment-induced changes in the expression levels of *OsTAA1*, *OsYUCCA1*, *OsPIN1* and *OsGH3.2*, *OsGH3.8* in Jigeng-88 was greater than that in Dongdao-4. Exogenous application of IAA resulted in different effects on the expression of these genes. Following the exogenous application of IAA, there was a significant increase in the expression levels of O*sTAA1*, *OsYUCCA1* and *OsPIN1* (Figure 7A–F), while there was no significant effect on the expression levels of *OsGH3.2* and *OsGH3.8* (Figure 7G–J). Similar to exogenous IAA application, exogenous TIBA application significantly reduced the expression levels of O*sTAA1*, *OsYUCCA1* and *OsPIN1* (Figure 7A–F) but had minimal effect on the expression levels of *OsGH3.2* and *OsGH3.8* (Figure 7G–J).

## 3. Discussion

The salinization of soil threatens agricultural productivity in many parts of the world, including north-eastern and north-western China [2,26]. Currently, these regions are commonly using large-scale irrigation methods to combat soil salinization, especially in rice-growing regions [26]. However, this technique may lead to low sodium levels and high pH levels in the soil. A soil with low sodium level and high pH level (alkaline stress) can negatively impact the growth and development of crops, but very little attention has been paid on this subject. As is well known, auxin play an important role in the regulation of abiotic stress in higher plants [27]. In this study, we therefore investigated whether auxin plays an important role in alkaline stress resistance in rice plants (using two genotypes with different tolerance to alkaline stress) through exogenous application of IAA/TIBA. Our results revealed that Dongdao-4 plants were more resistant to alkaline stress than Jigeng-88 plants, as evidenced by higher IAA concentration (Figure 1) and higher survival rates (Figure S1). A higher level of IAA concentration in plants may promote plant grow more strongly in stressful conditions [14,27], which was also demonstrated in our study by the fact that Dongdao-4 had larger shoots biomass (Figure 2). The enhancement of rice growth may account by the higher chlorophyll concentration and photosynthetic rates induced by the application of exogenous IAA (Figure 3). In addition, effective ROS detoxifying systems and a larger root system induced by the application of exogenous IAA may also contribute to the increased growth of rice seedlings grown in alkaline stress environment [28]. Previous studies have demonstrated that plants with a larger root system and higher antioxidant enzyme activities are capable of not only absorbing more nutrients from the soil for growth but are also better able to protect cell from oxidative stress [28]. Inconsistent with these findings, our research revealed that rice root parameters were less affected by alkaline stress when they were treated with exogenous IAA (Figure 4) and that the higher activities of antioxidant enzyme induced by exogenous application IAA enable plant accumulate less H_2_O_2_ and MDA (Figure 5 and Figure 6), thereby conferring tolerance to alkaline stress. The concentration of a plant’s auxin can be influenced by the processes of hormone synthesis, transport, and catabolism, as well as by the expression of genes that determine all these processes [14]. We, therefore, investigated the effect of alkaline stress on the expression of genes that are involved in the synthesis, transport, and catabolism of auxin. It was found that alkaline stress up-regulated the expression levels of genes controlling the transport and catabolism of auxin and down-regulated the expression levels of genes controlling the synthesis of auxin (Figure 7). While the application of exogenous IAA/TIBA significantly affected the expression of these related genes and, eventually, affected rice growth under alkaline stress by altering the concentration of auxin (Figure 7). In summary, our findings indicate that IAA plays an important role in alkaline stress tolerance by modulating root development and ROS detoxifying systems in rice plants.

Plant hormones play an important role in regulating plant responses to abiotic stress [14,29]. Nevertheless, only a few reports have been available up to now that evaluated the effect of plant hormones on the growth of plants under saline-alkaline stress conditions [8,9,30]. As an important plant hormone, auxin plays a significant role in regulating the growth of plants that are subjected to alkaline stress. In previous research, it has been demonstrated that when alkaline stress is applied to rice plants, alkaline-tolerant genotypes (Luna Suvarna) show higher root IAA concentrations than alkaline-sensitive genotypes (IR64) [15]. Similarly, the same results were also observed in malus rootstocks [19]. Generally, a higher level of IAA concentration in plant roots encourages root growth, which in turn promotes the development of the plant [27]. Mechanistically, the effect of growth hormone on root development under stress conditions is through the regulation of auxin homeostasis and transport, for example, by inhibiting IAA biosynthesis and promoting IAA efflux [27]. In accordance with these findings, our study also found that the formation and growth of root systems in rice plants were inhibited by alkaline stress, and exogenous IAA can alleviate this inhibition and promote root formation and elongation (Figure 4). In addition to IAA, recent studies have shown that higher levels of endogenous ABA or application of ABA increase rice’s tolerance to alkaline stress [8,31]. For instance, Liu et al. (2022) demonstrated that *OsABA8ox1* RNAi lines (higher concentration of ABA) were more tolerant to saline-alkaline stress than their wild-type counterparts, as reflected in the fact that their seedling survival and yield rates were higher [8]. Under alkaline stress conditions, higher levels of endogenous ABA or the application of ABA were found to activate the antioxidant defense system in a way that minimized the accumulation of ROS. This consequently resulted in the mitigation of alkali-induced root damage and seedling death [31]. In accordance with these results, our results also found that alkaline stress led to an increase in ABA in these two rice genotypes, and that the magnitude of the increase in Dongdao-4 plants was significantly greater than that of the increase in Jigeng-88 plants (Figure S2). As a consequence of the higher ABA concentration in Dongdao-4 plants, a greater tolerance to alkaline stress is also likely to be observed.

In plants, auxin levels are regulated by auxin biosynthesis and catabolism [14]. As one of the most important auxins of higher plants, IAA (Indole-3-acetic acid) is proposed to be synthesized by a Trp-(tryptophan)-dependent two-step pathway, which contributes significantly to the production of auxin [14]. During this pathway, Trp is first converted into IPA (indole-3-pyruvate) by the TAA1 (L-tryptophan pyruvate aminotransferase) family of aminotransferases and then into IAA by the *YUCCA* family of flavin monooxygenases [14]. In the biosynthesis of IAA, *OsYUCCA* plays a critical role as a rate-limiting enzyme. In rice, *OsYUCCA* is encoded by seven genes of which *OsYUCCA1* appear to play the most important role in producing IAA [32]. Generally, the expression levels of *OsYUCCA1* and *OsTAA1* was inhibited by multiple environmental stresses, such as salt stress, drought stress, osmotic stress, etc. [27,33,34]. In this study, we also showed that alkaline stress significantly inhibited the expression of IAA biosynthesis genes (*OsYUCCA1* and *OsTAA1*), with Jigeng-88 being significantly inhibited by alkaline stress than Dongdao-4 (Figure 7A–D). Furthermore, this study also found that the addition of exogenous IAA and TIBA altered the expression levels of *OsTAA1* and *OsYUCCA1* genes (Figure 7A–D). The exogenous addition of IAA alleviated the inhibition of *OsTAA1* and *OsYUCCA1* genes expression by alkaline stress, whereas the exogenous addition of TIBA exacerbated the inhibition (Figure 7A–D). IAA is synthesized at the root and stem tips and then transported by an IAA transporter to the site of action [14]. IAA transporters are encoded by the *OsPIN* gene, whose expression can be inhibited by various environmental stresses, such as salt stress, cold stress, drought stress, etc. [14,27,35]. There is evidence that increasing the expression levels of the *OsPIN* gene can increase the plant’s resistance to environmental stresses [14,35]. In accordance with the above results, we found that the expression levels of *OsPIN1* was significantly inhibited by alkaline stress, with Jigeng-88 being significantly more inhibited than Dongdao-4 (Figure 7E,F). Moreover, the exogenous addition of IAA alleviated the alkaline stress-induced inhibition of *OsPIN1* expression, whereas the exogenous addition of TIBA, on the other hand, exacerbated the alkaline stress-induced inhibition of *OsPIN1* expression (Figure 7E,F). Generally, IAA catabolism is thought to determine the amount of active IAA in a given cell and is therefore important for many developmental processes [14]. It is found that IAA can be converted to ester conjugates with sugars by UGTs (UDP-glucose transferases) or to amide conjugates with amino acids by GH3 amino acid conjugate synthetases [14]. The expression of *OsGH*3 gene family is affected by a variety of different stress factors and is associated with auxin-deficient traits and resistance to abiotic stresses [14,36]. In rice, 13 GH3 genes have been identified, and all *OsGH3* genes play an important role in the response to abiotic stress [36]. As an example, the expression of *OsGH3.2* and *OsGH3.8* is significantly induced by salt stress, indicating that *OsGH3.2* and *OsGH3.8* play important roles during salt stress [36]. Similarly, our results showed that alkaline stress significantly increased the expression of IAA catabolism genes (*OsGH3.2* and *OsGH3.8*), with Jigeng-88 being significantly enhanced by alkaline stress than Dongdao-4 (Figure 7G–J). In contrast to IAA biosynthesis (*OsYUCCA1* and *OsTAA1*) and transporter genes (*OsPIN*), it was found that the exogenous addition of IAA and TIBA did not significantly alter the expression of *OsGH3* genes in this study (Figure 7).

As part of the process of photosynthesis, chlorophyll plays a crucial role in absorbing light and converting it into chemical energy that can be used by plants. Whenever there is chlorophyll degradation, plants are unable to achieve their full photosynthetic potential, which adversely affects their growth and development, and also their ability to thrive [37]. In general, environmental stress causes a decrease in the foliar chlorophyll concentration, as well as a decrease in the plant’s photosynthetic rate. It was found that exogenous application of IAA could increase the content of chlorophyll a and chlorophyll b, thus increasing the photosynthetic rate [16]. The presence of auxin enhances photosynthetic rate by increasing the density of leaf veins, whose well-organized placement drives the increase in photosynthetic capacity of leaf surfaces [38]. Enhancement of photosynthetic rate may also result in greater stomatal aperture induced by exogenous application of IAA under stress condition [22,23]. Additionally, IAA and its precursors, including L-tryptophan (Trp) and indole (Ind), increase the amount of mineral nutrients in plant leaves, such as magnesium which also play important role in chlorophyll synthesis [23,39]. In summary, higher endogenous IAA concentration or application of exogenous IAA will enhance chlorophyll synthesis, widened stomatal aperture, increase photosynthesis rate, thereby increase the tolerance to alkaline stress in rice plants. In agreement with these results, our study also found that IAA application mitigated the alkaline stress-induced reduction of foliar chlorophyll concentration in both genotypes of rice, thereby mitigating the negative effect of alkaline stress on photosynthetic rate and plant growth (Figure 3). In addition, it has been shown that the application of exogenous IAA under alkaline stress can protect the photosystem by enhancing proton dynamics and reducing proton gradients [40] and, therefore, improve photosynthetic rates in both genotypes of rice plants under alkaline stress (Figure 3).

Auxin is an important plant hormone well-known for controlling root development [41]. Under stress conditions, the accumulation and distribution of auxin in roots will be altered, thus affecting root morphology [27,42]. Among these studies, the mechanism of the effect of salt stress on root architecture is well studied [27]. Several studies have indicated that salt stress alters the accumulation and distribution of auxin, causing changes in the root architecture by inhibiting the growth of primary and lateral roots [42]. In similar to salt stress, root IAA concentration was decreased by alkaline stress, which resulting in decreasing of the adventitious root number, total root length, root surface area and dry root biomass [11]. Saini et al. (2021) have found that Luna Suvarna (salt tolerant cultivar) plants have a higher root IAA concentration than IR64 (a salt sensitive cultivar), which confers it greater root length, adventitious root number, and dry root biomass than IR64 plants [15]. Exogenous IAA treatment promote root growth, enable plants have a bigger root system than that plant under control condition [15,19]. Under stress conditions, a bigger root system may contribute to nutrition acquisition of plants [23]. In agreement with these results, our study found that alkaline-induced decrease in the adventitious root number, total root length, root surface area and dry root biomass in Dongdao-4 was less than that in Jigeng-88, and IAA application mitigated the alkaline stress-induced reduction of these root parameters in both rice genotypes (Figure 4). These findings imply that a higher endogenous IAA concentration or application of exogenous IAA can contribute to the development of a more extensive root system in rice seedlings, which may promote nutrition acquisition, thereby enhancing tolerance to alkaline stress.

Normally, reactive oxygen species are in dynamic equilibrium [28]. However, if plants are subjected to abiotic stresses, such as herbicides, pesticides, saline stress, or alkaline stress, they accumulate in large quantities [23,43,44]. The large quantity of reactive oxygen species that accumulate over time will damage the membrane structure of the plant and will affect the plant’s normal metabolism as a result of the peroxidation of nucleic acids, proteins, and lipids [28]. At the same time, reactive oxygen species also damage the selectivity of the membrane of the cell, leading to an increase in membrane permeability and the occurrence of membrane lipid peroxidation, which is eventually likely to cause plant damage or even death [28]. Moreover, free radicals act on the lipid peroxidation process, and the end product of this oxidation process is MDA, which can crosslink and polymerize with proteins, nucleic acids, and other living macromolecules, making them no longer active [45]. In this study, it was found that alkaline stress stimulated the formation of H_2_O_2_ and MDA in both rice genotypes, and following the application of exogenous IAA, there was a decrease in the content of H_2_O_2_ and MDA in both rice genotypes (Figure 5). In recent years, it has been reported that IAA plays a very important role in the activation of antioxidant enzymes under stress conditions [46]. Whenever IAA has been applied to plants, the activities of SOD, CAT, and POD were increased [16,46], the ability to scavenge ROS was increased, as well as the antioxidant capacity of plants was improved. In the current study, we have also found that exogenous IAA treatment increased the activity of SOD, CAT, and POD under alkaline stress conditions (Figure 6). The findings in this study are in accordance with those found in the existing literature. Using exogenous IAA may be an effective way of enhancing the activity of antioxidant enzymes in rice seedlings under alkaline conditions, thus enhancing their ability to scavenge ROS and increasing their antioxidant effects.

In summary, this study demonstrated that Dongdao-4, an elite rice genotype that was bred in saline-alkaline soil in northern China was more tolerant to alkaline stress. When grown in alkaline conditions, Dongdao-4 plants can accumulate more IAA than Jigeng-88 plants and confers on it a larger root system and ROS detoxifying systems. In addition, application of exogenous IAA mitigates the effect of alkaline stress on root parameters and ROS accumulation. An extensive root system, as well as ROS detoxifying systems, strengthened with IAA may enable rice plant have better able to tolerate alkaline stress. These findings will be valuable for our understanding of the physiological mechanisms of rice plants in responses to alkaline stress.

## 4. Materials and Methods

### 4.1. Plant Materials and Germination Treatments

In this study, the varieties of rice Dongdao-4 and Jigeng-88 from the species *Oryza sativa* L. ssp. Japonica was utilized. After 2 days of germination in tap water at 37 °C, the seeds were put on moist tissue paper and kept in the dark at 30 °C for 2 days. The seedlings were then moved to a solution containing (mM): 1.425 NH_4_NO_3_, 0.42 NaH_2_PO_4_, 0.510 K_2_SO_4_, 0.998 CaCl_2_, 1.643 MgSO_4_, 0.168 Na_2_SiO_3_, 0.125 Fe-EDTA, 0.019 H_3_BO_3_, 0.009 MnCl_2_, 0.155 CuSO_4_, 0.152 ZnSO_4_, and 0.075 Na_2_MoO_4_ and were cultivated in a growth chamber with a 14-hour photoperiod at a constant temperature of 30 °C/22 °C (day/night) and relative humidity of approximately 70%.

In order to determine the tolerance of these two rice genotypes to alkaline stress, the rice plants were exposed to a nutritious solution for 2 weeks, and then half of the plants were moved to a solution containing 20 mM NaHCO_3_ and a pH of 8.5 for 5 days. To determine the effects of IAA and TIBA on the growth of rice plants under alkaline stress, two-week-old seedlings were transferred to an alkaline stress treatment solution containing 6 μM IAA and 30 μM TIBA for 5 days. For both the control and treatment media, the pH was adjusted and the solution was changed every 2 days.

### 4.2. Measurements of Plant Growth

After treatments, the plants’ height was measured with a meter ruler. To determine the rice seedlings’ dry biomass, the shoots and roots were plucked and oven-dried at 75 °C for 2 days until their weight was constant.

In order to examine root morphological parameters, an Epson digital scanner (Expression 10000XL, Epson (China) Co., Ltd., Beijing, China) was used to scan roots, which were then processed using the WinRHIZO/WinFOLIA program (Regent Instruments Inc., Québec City, QC, Canada).

### 4.3. Measurements of Chlorophyll (CHL) Concentration

In this study, the chlorophyll concentration was determined as described by Sharma et al., (2012) [47]. To determine the concentration of CHL in rice leaves, freshly harvested leaves were weighed, distilled, and extracted with 95% (*v*/*v*) aqueous ethanol. The absorbance of the supernatant was measured using wavelengths of 663 nm and 645 nm. Based on the calculated chlorophyll content per gram of fresh mass, the total chlorophyll content is 8.02 A663 + 20.21 A645 mg.

### 4.4. Measurements of Photosynthetic Characteristics

A portable photosynthesis system (LI-6400 XT) equipped with an LED leaf cuvette (Li-Cor, 146, Lincoln, NE, USA) was used to measure the photosynthetic rate of rice seedlings between 8:30 and 11:30. In the chamber, leaves were artificially illuminated by 6400-02B LEDs mounted on the sensor head and were illuminated continuously for 1000 mol m^−2^ s^−1^ photosynthetic photon flux density and 500 moL CO_2_ mol ambient CO_2_ concentration. The photosynthetic rates of rice plants were evaluated individually for each treatment by evaluating a minimum of 15 plants per treatment.

### 4.5. Determination of H_2_O_2_

In this study, hydrogen peroxide was measured in accordance with Alexieva et al. (2001) with some modification [48]. In brief, 1 g of samples were ground in 2 mL of 0.1% trichloroacetic acid (TCA), and 8 mL of TCA was then added to rinse the mortar and pestle. Following this, the sample was centrifuged at 10,000× *g* at 4 °C for 20 min. The supernatant (1 mL) was then diluted with 1 mL of potassium phosphate buffer pH (7.0) and 2 mL of 1 M KI. After mixing and dark reaction at room temperature for 60 min, the OD value was measured at 390 nm. To calculate the amount of hydrogen peroxide in the mixture, a standard curve of known hydrogen peroxide concentrations was prepared.

### 4.6. Determination of Malondialdehyde (MDA)

The levels of malondialdehyde in rice leaves were determined following the procedure described by [48]. In brief, the leaves of rice were weighed and homogenized in a 10% TCA solution in 5 mL, after which they were centrifuged for 10 min at 10,000× *g*. Following centrifugation, 2 mL of supernatant was combined with 2 mL of 10% TCA containing 0.6% thiobarbituric acid. After incubation at 95 °C for 30 min, the reaction was stopped using an ice bath. The solution’s absorbance was measured at three different wavelengths: 450, 532, and 600 nm.

### 4.7. Determination of Peroxidase, Superoxide Dismutase, and Catalase Activity

Rice leaves were ground thoroughly to a weight of approximately 0.5 g in a pH 7.8 potassium phosphate buffer (50 mM) with 1% polyvinylpyrrolidone using a cold mortar and pestle. Following homogenization, the homogenate was centrifuged at 15,000× *g* at temperature of 4 °C for 20 min. A crude enzyme extraction was obtained from the supernatant. The activities of three enzymes, superoxide dismutase (SOD; EC 1.15.1.1), peroxidase (POD; EC 1.11.1.7), and catalase (CAT; EC 1.11.1.6), were evaluated using the protocols described by Giannopolitis and Ries (1977) [49], Gasper et al. (1975) [50] and Aebi (1984) [51], respectively.

### 4.8. RNA Isolation and Real-Time RT-PCR

The activity of peroxidase, superoxide dismutase, and catalase was determined following the procedures described by Li et al. (2016) [11]. Briefly, total RNA was extracted using Trizol (Invitrogen, Carlsbad, CA, USA) as the extraction reagent and treated with DNase I (Promega, Madison, WI, USA) as the DNase treatment protocol (USA). A 20-L volume of total RNA was reverse transcribed into first-strand cDNA with M-MLV reverse transcriptase (Promega). In an optical 96-well plate, real-time PCR was performed utilizing an Applied Biosystems Step One TM Real-Time PCR apparatus. During each reaction, 5 L of diluted cDNA was added to 12.5 L of SYBR GreenER qPCR SuperMix Universal (Invitrogen), 0.5 L of Rox Reference Dye, 1 L of 10 M forward and reverse primers, and 5 L of sterile water. During the heat cycle, 95 °C was maintained for 10 min, followed by 40 cycles of 95 °C for 30 s, 60 °C for 30 s, and 72 °C for 30 s. Table S1 provides a list of the primers used for each gene. In order to ensure internal quality control, Actin (GenBank accession number AB047313) was employed as a control. In order to determine the relative level of expression, the comparative CT method was used.

### 4.9. Statistical Analysis

A variance analysis was conducted using SAS statistical software. The significance of the differences between treatments was determined using a student’s *t*-test.

## 5. Conclusions

In this study, we present experimental evidence demonstrating that IAA and TIBA can modulate the responses of rice seedlings to alkaline stress. More specifically, we demonstrate that rice seedlings treated with IAA are more resilient to alkaline stress. Our results also suggest that rice plants may be better able to tolerate alkaline stress when they have an extensive root system, as well as ROS detoxifying systems, strengthened with IAA. Moreover, these findings provide important mechanistic insights for our understanding of rice plants when they are subjected to alkaline stress conditions.

## Supplementary Materials

The following supporting information can be downloaded at: https://www.mdpi.com/article/10.3390/ijms232314817/s1, Figure S1: Effects of alkaline stress on Dongdao-4 and Jigeng-88 seedlings; Figure S2: ABA concentrations of (A) shoots and (B) roots of Dongdao-4 and Jigeng-88 seedlings grown in normal and saline-alkaline stress conditions; Table S1: Primers used for quantitative real-time PCR in this study.

## References

[1] Vinod K.K., Krishnan S.G., Babu N.N., Nagarajan M., Singh A.K., Ahmad P., Azooz M.M., Prasad M.N.V. (2013). Improving salt tolerance in rice: Looking beyond the conventional. Salt Stress in Plants: Signalling Omics and Adaptations.

[2] Haefele S.M., Nelson A., Hijmans R.J. (2014). Soil quality and constraints in global rice production. Geoderma.

[3] Munns R., Tester M. (2008). Mechanisms of Salinity Tolerance. Annu. Rev. Plant Biol..

[4] Mohammad Z., Shabbir A.S., Lee H. (2018). Guideline for Salinity Assessment, Mitigation and Adaptation Using Nuclear and Related Techniques.

[5] Huang L.H., Liang Z.W., Suarez D.L., Wang Z.C., Wang M.M., Yang H.Y., Liu M. (2015). Impact of cultivation year, nitrogen fertilization rate and irrigation water quality on soil salinity and soil nitrogen in saline-sodic paddy fields in Northeast China. J. Agric. Sci..

[6] León-Lorenzana A.S., Delgado-Balbuena L., Domínguez-Mendoza C., Navarro-Noya Y.E., Luna-Guido M., Dendooven L. (2017). Reducing salinity by flooding an extremely alkaline and saline soil changes the bacterial community but its effect on the archaeal community is limited. Front. Microbiol..

[7] Kobayashi N.I., Yamaji N., Yamamoto H., Okubo K., Ueno H., Costa A., Tanoi K., Matsumura H., Fujii-Kashino M., Horiuchi T. (2017). OsHKT1;5 mediates Na^+^ exclusion in the vasculature to protect leaf blades and reproductive tissues from salt toxicity in rice. Plant J..

[8] Liu X., Xie X., Zheng C., Wei L., Li X., Jin Y., Zhang G., Jiang C., Liang Z. (2022). RNAi-mediated suppression of the abscisic acid catabolism gene *OsABA8ox1* increases abscisic acid content and tolerance to saline–alkaline stress in rice (*Oryza sativa* L.). Crop J..

[9] Xu Z., Wang J., Zhen W., Sun T., Hu X. (2022). Abscisic acid alleviates harmful effect of saline-alkaline stress on tomato seedlings. Plant Physiol. Biochem..

[10] Rasheed R., Ashraf M.A., Ahmad S.J.N., Parveen N., Hussain I., Bashir R. (2022). Taurine regulates ROS metabolism, osmotic adjustment, and nutrient uptake to lessen the effects of alkaline stress on *Trifolium alexandrinum* L. plants. S. Afr. J. Bot..

[11] Li Q., Yang A., Zhang W.H. (2016). Efficient acquisition of iron confers greater tolerance to saline-alkaline stress in rice (*Oryza sativa* L.). J. Exp. Bot..

[12] An Y., Yang X.X., Zhang L., Zhang J., Du B., Yao L., Li X.T., Guo C. (2020). Alfalfa *MsCBL4* enhances calcium metabolism but not sodium transport in transgenic tobacco under salt and saline-alkali stress. Plant Cell Rep..

[13] Shi D., Yin S., Yang G., Zhao K. (2002). Citric acid accumulation in an alkali-tolerant plant *Puccinellia tenuiflora* under alkaline stress. Acta Bot. Sin..

[14] Naser V., Shani E. (2016). Auxin response under osmotic stress. Plant Mol. Biol..

[15] Saini S., Kaur N., Marothia D., Singh B., Singh V., Gantet P., Pati P.K. (2021). Morphological analysis, protein profiling and expression analysis of auxin homeostasis genes of roots of two contrasting cultivars of rice provide inputs on mechanisms involved in rice adaptation towards salinity stress. Plants.

[16] Gong B., Miao L., Kong W., Bai J.G., Wang X., Wei M., Shi Q. (2014). Nitric oxide, as a downstream signal, plays vital role in auxin induced cucumber tolerance to sodic alkaline stress. Plant Physiol. Biochem..

[17] Huang J., Wu Q., Jing H.K., Shen R.F., Zhu X.F. (2022). Auxin facilitates cell wall phosphorus reutilization in a nitric oxide-ethylene dependent manner in phosphorus deficient rice (*Oryza sativa* L.). Plant Sci..

[18] Wu J., Cao J., Su M., Feng G., Xu Y., Yi H. (2019). Genome-wide comprehensive analysis of transcriptomes and small RNAs offers insights into the molecular mechanism of alkaline stress tolerance in a citrus rootstock. Hortic. Res..

[19] Wen T., Dong L., Wang L., Ma F., Zou Y., Li C. (2018). Changes in root architecture and endogenous hormone levels in two Malus rootstocks under alkali stress. Sci. Hortic..

[20] He Y., Zhang T., Sun Y., Wang X., Cao Q., Fang Z., Chang M., Cai Q., Lou L. (2022). Exogenous IAA alleviates arsenic toxicity to rice and reduces arsenic accumulation in rice grains. J. Plant Growth. Regul..

[21] Mehmood A., Hussain A., Irshad M., Khan N., Hamayun M., Ismail, Afridi S.G., Lee I.-J. (2018). IAA and flavonoids modulates the association between maize roots and phytostimulant endophytic Aspergillus fumigatus greenish. J. Plant Interact..

[22] Shiraz M., Sami F., Siddiqui H., Yusuf M., Hayat S. (2020). Interaction of auxin and nitric oxide improved photosynthetic efficiency and antioxidant system of brassica juncea plants under salt stress. J. Plant Growth Regul..

[23] Mir A.R., Siddiqui H., Alam P., Hayat S. (2020). Foliar spray of Auxin/IAA modulates photosynthesis, elemental composition, ROS localization and antioxidant machinery to promote growth of Brassica juncea. Physiol. Mol. Biol. Plants.

[24] Kaya C., Tuna A.L., Dikilitas M., Cullu M.A. (2010). Responses of some enzymes and key growth parameters of salt-stressed maize plants to foliar and seed applications of kinetin and indole acetic acid. J. Plant Nutr..

[25] Kinoshita N., Wang H., Kasahara H., Liu J., Macpherson C., Machida Y., Kamiya Y., Hannah M.A., Chua N.H. (2012). IAA-Ala Resistant3, an evolutionarily conserved target of miR167, mediates Arabidopsis root architecture changes during high osmotic stress. Plant Cell.

[26] Liu M., Yang J., Li X., Liu G., Yu M., Wang J. (2012). Distribution and dynamics of soil water and salt under different drip irrigation regimes in northwest China. Irrig. Sci..

[27] Ribba T., Garrido-Vargas F., O’Brien J.A. (2020). Auxin-mediated responses under salt stress: From developmental regulation to biotechnological applications. J. Exp. Bot..

[28] Dumanovic J., Nepovimova E., Natic M., Kuca K., Jacevic V. (2020). The significance of reactive oxygen species and antioxidant defense system in plants: A concise overview. Front. Plant Sci..

[29] Rhaman M.S., Imran S., Rauf F., Khatun M., Baskin C.C., Murata Y., Hasanuzzaman M. (2020). Seed priming with phytohormones: An effective approach for the mitigation of abiotic stress. Plants.

[30] Zhou C., Zhu L., Xie Y., Li F., Xiao X., Ma Z., Wang J. (2017). Bacillus licheniformis SA03 confers increased saline-Alkaline tolerance in chrysanthemum plants by induction of abscisic acid accumulation. Front. Plant Sci..

[31] Liu X., Zhang H., Jin Y., Wang M., Yang H., Ma H., Jiang C., Liang Z. (2019). Abscisic acid primes rice seedlings for enhanced tolerance to alkaline stress by upregulating antioxidant defense and stress tolerance-related genes. Plant Soil.

[32] Yamamoto Y., Kamiya N., Morinaka Y., Matsuoka M., Sazuka T. (2007). Auxin biosynthesis by the *YUCCA* genes in rice. Plant Physiol..

[33] Da Costa M.V.J., Ramegowda V., Sreeman S., Nataraja K.N. (2021). Targeted phytohormone profiling identifies potential regulators of spikelet sterility in rice under combined drought and heat stress. Int. J. Mol. Sci..

[34] Lee S., Huang W. (2013). Osmotic stress stimulates shoot organogenesis in callus of rice (*Oryza sativa* L.) via auxin signaling and carbohydrate metabolism regulation. Plant Growth Regul..

[35] Khan M.A., Asaf S., Khan A.L., Adhikari A., Jan R., Ali S., Imran M., Kim K.-M., Lee I.-J. (2020). Plant growth-promoting endophytic bacteria augment growth and salinity tolerance in rice plants. Plant Biol..

[36] Kong W., Zhong H., Deng X., Gautam M., Gong Z., Zhang Y., Zhao G., Liu C., Li Y. (2019). Evolutionary analysis of *GH3* genes in six *Oryza* species/subspecies and their expression under salinity stress in *Oryza sativa* ssp. japonica. Plants.

[37] Turan S., Tripathy B.C. (2015). Salt-stress induced modulation of chlorophyll biosynthesis during de-etiolation of rice seedlings. Physiol. Plant.

[38] McAdam S.A.M., Eleouet M.P., Best M., Brodribb T.J., Murphy M.C., Cook S.D., Dalmais M., Dimitriou T., Gelinas-Marion A., Gill W.M. (2017). Linking Auxin with Photosynthetic Rate via Leaf Venation. Plant Physiol..

[39] San-Francisco S., Houdusse F., Zamarreño A.M., Garnica M., Casanova E., García-Mina J.M. (2005). Effects of IAA and IAA precursors on the development, mineral nutrition, IAA content and free polyamine content of pepper plants cultivated in hydroponic conditions. Sci. Hortic..

[40] Su L., Xie J., Wen W., Li J., Zhou P., An Y. (2020). Interaction of zinc and IAA alleviate aluminum-induced damage on photosystems via promoting proton motive force and reducing proton gradient in alfalfa. BMC Plant Biol..

[41] Overvoorde P., Fukaki H., Beeckman T. (2010). Auxin control of root development. Cold Spring Harb. Perspect. Biol..

[42] Julkowska M.M., Hoefsloot H.C., Mol S., Feron R., de Boer G.J., Haring M.A., Testerink C. (2014). Capturing Arabidopsis root architecture dynamics with ROOT-FIT reveals diversity in responses to salinity. Plant Physiol..

[43] Vaidyanathan H., Sivakumar P., Chakrabarty R., Thomas G. (2003). Scavenging of reactive oxygen species in NaCl-stressed rice (*Oryza sativa* L.)—Differential response in salt-tolerant and sensitive varieties. Plant Sci..

[44] Bailey D.C., Todt C.E., Burchfield S.L., Pressley A.S., Denney R.D., Snapp I.B., Negga R., Traynor W.L., Fitsanakis V.A. (2018). Chronic exposure to a glyphosate-containing pesticide leads to mitochondrial dysfunction and increased reactive oxygen species production in Caenorhabditis elegans. Environ. Toxicol. Pharmacol..

[45] Gaweł S., Wardas M., Niedworok E., Wardas P. (2004). Malondialdehyde (MDA) as a lipid peroxidation marker. Wiad. Lek..

[46] Agami R.A., Mohamed G.F. (2013). Exogenous treatment with indole-3-acetic acid and salicylic acid alleviates cadmium toxicity in wheat seedlings. Ecotoxicol. Environ. Saf..

[47] Sharma P., Bhatt D., Zaidi M.G., Saradhi P.P., Khanna P.K., Arora S. (2012). Silver nanoparticle-mediated enhancement in growth and antioxidant status of Brassica juncea. Appl. Biochem. Biotechnol..

[48] Alexieva V., Sergiev I., Mapelli S., Karanov E. (2001). The effect of drought and ultraviolet radiation on growth and stress markers in pea and wheat. Plant Cell Environ..

[49] Giannopolitis C.N., Ries S.K. (1977). Superoxide DismutasesI. Occurrence in higher plants. Plant Physiol..

[50] Gasper T., Penel C., Greppin H. (1975). Peroxidase and isoperoxidase in relation to root and flower formation. Plant Biochem. J..

[51] Aebi H. (1984). Catalase in vitro. Methods Enzymol..

