# Supplementary material for: IAA Plays an Important Role in Alkaline Stress Tolerance by Modulating Root Development and ROS Detoxifying Systems in Rice Plants
Source: Int J Mol Sci. 2022 Nov 26;23(23):14817. doi: 10.3390/ijms232314817 (PMC9740826; doi:10.3390/ijms232314817)
Supplement: Supplementary file 1 [file ijms-23-14817-s001.zip › ijms-2037394-supplementary.pdf]

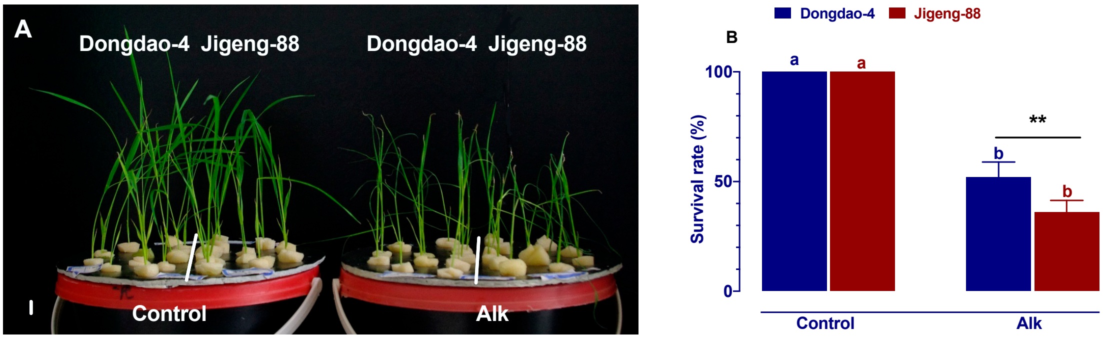

Figure S1. Effects of alkaline stress on Dongdao-4 and Jigeng-88 seedlings. (A) Growth performance. (B) Survival rate. One-week-old rice seedlings grown in normal culture solution were transferred to culture solution supplemented with 20 mM $NaHCO_3$ and a pH of 8.5 for 2 days. Survival rate was measured after treatment with alkaline stress for one week. Alk=alkaline stress. Bars=10 cm. Data are means±SE ($n \geq 5$). Means with different letters are significantly different between control and alkaline stress of the same genotype ($P<0.05$). Asterisks indicate significant differences between different genotypes within the same treatment (**$P<0.01$).

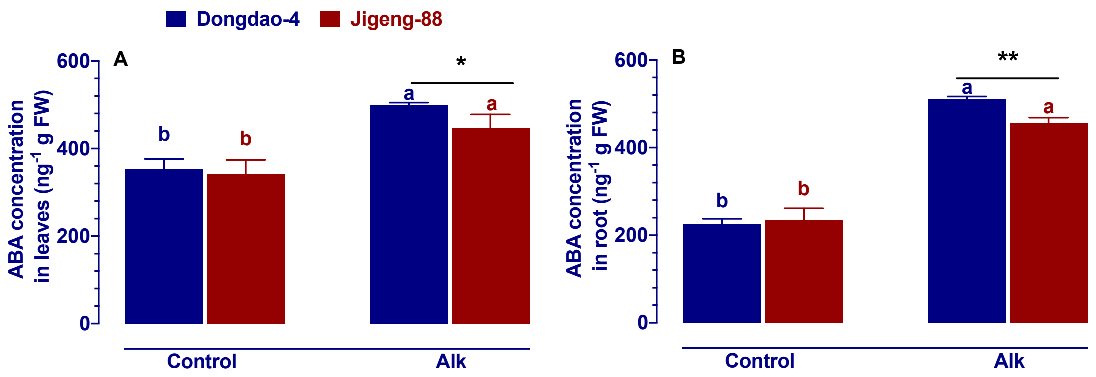

Figure S2. ABA concentrations of (A) shoots and (B) roots of Dongdao-4 and Jigeng-88 seedlings grown in normal and saline-alkaline stress conditions. Two-week-old rice seedlings grown in normal culture solution were transferred to culture solution supplemented with 20 mM $NaHCO_3$ and a pH of 8.5 for 5 days. Alk=alkaline stress. Bars=10 cm. Data are means±SE (*n*≥5). Means with different letters are significantly different between control and alkaline stress of the same genotype (*P*<0.05). Asterisks indicate significant differences between different genotypes within the same treatment (**P*<0.05, ***P*<0.01).

Table S1. Primers used for quantitative real-time PCR in this study

**Gene**	**Forward primer 5' → 3'**	**Reverse primer 5' → 3'**
OsYUCCA1	TCATCGGACGCCCTCAACGGGTCGC	GGCAGAGCAAGATTATCAGTC
OsTAA1	GTCCTGCCAAATAGGTTCTCA	TCACGCAGGCACTGATCTAC
OsPIN1	AGTACAAAGCTTGGGGGGAC	ATCTCTTGTCAGAATCGGCG
OsGH3.2	GCTAGACGACACAACGATATATAGCC	CTGATGCCCTCTGCCTTAAACAC
OsGH3.8	GTCCGAATAGTCGGTCAAATCC	TGCCACTAACTGACAGAGTTGACAG
Actin	ACCACAGGTATTGTGTTGGACTC	AGAGCATATCCTTCATAGATGGG
